# Integrated CO_2_ Laser Treatment and Cognitive–Behavioral Therapy for Postadolescent Acne Excoriée: Clinical Outcomes From a Case Series

**DOI:** 10.1111/jocd.70722

**Published:** 2026-02-15

**Authors:** Julian Penev, Milla Balabanova, Mohammad Jafferany

**Affiliations:** ^1^ Department of Infectious Diseases, Parasitology and Dermatovenerology Medical University Varna Varna Bulgaria; ^2^ Department of Psychiatry and Behavioral Sciences Central Michigan University College of Medicine Michigan USA

**Keywords:** acne excoriée, cognitive behavioral therapy, excoriation, fractional CO_2_ laser, psychodermatology

## Abstract

**Background:**

Self‐inflicted skin injury is a significant yet often underrecognized contributor to acne progression, particularly in postadolescent populations. It not only perpetuates lesions but also triggers inflammatory cascades, including sebaceous gland epithelialization and cystic transformation. Postadolescent acne excoriée poses unique therapeutic challenges due to the interplay of dermatological and psychological factors.

**Aims:**

To evaluate the effectiveness of a combined psychodermatological treatment—CO_2_ laser ablation and cognitive–behavioral therapy (CBT)—in managing chronic, treatment‐resistant postadolescent acne excoriée.

**Methods:**

A case series of three patients with refractory postadolescent acne excoriée was conducted. Treatment involved CO_2_ laser ablation to remove obstructed sebaceous glands, combined with CBT targeting maladaptive excoriation behaviors.

**Results:**

All patients achieved rapid and sustained clinical improvement. The combined approach disrupted the pathomimetic cycle, eliminated visible obstructed sebaceous glands, and reduced compulsive skin manipulation.

**Conclusion:**

Integrating CO_2_ laser ablation with CBT offers a dual benefit of addressing both the physical lesions and psychological drivers of acne excoriée. Multidisciplinary collaboration is essential for effective management of complex psychocutaneous disorders.

## 1 Introduction

Acne remains a prevalent dermatological disease whose complex etiology and pathogenesis continue to elude scientific consensus. This persistent uncertainty likely stems from the non‐holistic understanding of the disease. Often dismissed as a minor teenage problem, chronic post‐adolescent and treatment‐resistant acne poses significant therapeutic challenges. For many patients with prolonged, post‐adolescent acne, the psychological wounds run far deeper than the physical lesions. What begins as a common skin disorder frequently evolves into a complex psychodermatological phenomenon that reshapes lives, relationships, and self‐perception in profound ways. The psychosocial toll of acne manifests through canceled life events, avoided social interactions, declined career opportunities, academic setbacks, and strained relationships, to mention just a few. Beyond the visible lesions, acne impairs psychological well‐being, erodes self‐esteem, and diminishes quality of life, with affected individuals demonstrating elevated rates of anxiety, stress, depression, physical discomfort, and disturbing prevalence of suicidal ideation [1, 2, 3, 4, 5, 6, 7]. There is evidence that the increased secretion of corticotropin‐releasing hormone, glucocorticoids, and adrenal androgens [8] leads to enhanced conversion of androgen precursors into testosterone and an increase in sebaceous hyperplasia [9]. Acne manifests as a psychosomatic and psychobehavioral condition, with many cases of persistent post‐adolescent acne demonstrating notable psychiatric comorbidity. This is especially evident in acne excoriée where patients exhibit pathological skin manipulation behaviors, including compulsive picking, squeezing, and rubbing of lesions, as well as instrument‐assisted trauma (needle puncturing) with the use of magnifying devices (5×, 7× magnifying mirrors). This type of acne belongs to stress‐aggravated psychodermatological disorders and coexists with a wide range of psychiatric disorders [6]. It is characterized by the repetitive and uncontrollable urge to pick, scratch, or rub acne lesions. This compulsive behavior often worsens the condition, leading to scarring, persistent inflammation, and impaired healing. The compulsive skin‐picking could be considered a hallmark behavior in acne excoriée, for the psychobehavioral component is not merely a secondary factor—it is a pathogenic driver.

We followed DSM‐5 criteria for excoriation (Skin‐Picking) disorder, as follows:

- Recurrent picking → lesions and inflammation (all cases);
- Repeated failed attempts to stop (“tries not to pick” and “avoids mirror”);
- Significant distress and functional impairment (social withdrawal, avoidance of professional opportunities, and dependence on makeup).
- The term “treatment‐resistant acne excoriée” was diagnosed with acne duration > 2 years;
- Failure to respond to ≥ 2 systemic therapies (e.g., antibiotics and isotretinoin);
- Presence of ritualistic excoriation with hyper‐focus (use of magnifying mirrors);
- Psychosocial maladaptation (shame, avoidance, and low self‐esteem).

These criteria complement the dermatological definition, where acne excoriée is a subtype of neurotic excoriation with a focus on acne lesions [10, 11].

## 2 Methods/Case Series Presentation

We report three cases of chronic post‐adolescent acne excoriée successfully treated with a combined approach of psychotherapy and laser‐assisted drainage of cystic, epithelialized sebaceous glands.

### 2.1 Patient Profile

All patients had treatment‐resistant acne excoriée. Consent was obtained from patients for possible publication. This study was exempt from mandatory institutional ethics approval as no experimental interventions were implemented. The methods used (low‐power CO_2_ laser and CBT) are part of standard clinical practice. Therapy was prescribed for medical indications, not for research purposes.

### 2.2 Dual‐Modality Treatment

Psychotherapy targeted compulsive picking, while CO_2_ laser therapy used low‐power (3–4 W) super pulse mode with a focused 250 μm beam to perforate and evacuate clogged sebaceousglands. This perforation destroys the gland, liquefies the sebum, making it easy to evacuate using a comedone extractor loop.

The choice of 250 μm spot size and 3–4 W in the superpulse mode is targeted for deep micro‐drainage without carbonization and minimal pain. 250 μm enables precise perforation of individual follicles and cysts without damaging surrounding tissue. 3–4 W in superpulse delivers controlled thermal energy sufficient to create a microchannel for atraumatic evacuation of sebaceous content, minimizing collateral damage. The goal is destruction of the inflammatory source—the epithelialized sebaceous gland (transformed into a cyst by chronic inflammation)—thereby breaking the pathogenic cycle.

Our psychotherapeutic intervention was structured, evidence‐based, and fully aligned with established protocols: Session 0: clinical interview, functional behavioral analysis, and treatment plan formulation. Sessions 1–3 and beyond: Habit Reversal Training (HRT): awareness of triggers + competing response (“touch only the untreated side”). CBT: cognitive restructuring (“my blood is not toxic”); Mindfulness & ERP: urge acceptance without action; Maintenance: relapse prevention.

### 2.3 Case Report 1

Patient is a 35‐year‐old woman with acne persisting for over 10 years despite multiple systemic, topical, and cosmetic treatments, including a 6‐month course of oral antibiotics, all with poor results. She maintained strict hygiene including washing her face three times daily with antibacterial products but regularly picked her acne under a magnifying mirror. She admitted to compulsively squeezing both non‐inflamed papules and pustules, often keeping her hand on her chin to search for “something to squeeze.”

Given the suspected psychiatric component, treatment targeted self‐excoriation through psychotherapy and included radical removal of epithelialized sebaceous glands. This was achieved through laser ablative perforation and evacuation of residual sebum content using a comedone extractor loop. A maximally focused beam (250 μm) was used at low laser output power (3–4 W) in the superpulse mode.

During the first laser session, all visible papules were removed on one side of the face to let the patient assess effectiveness. Psychological therapy began in parallel to address and stop self‐excoriation. She was advised to discontinue previous treatments, stop cosmetic products, and limit skin care to water only. Positive results from the initial session reduced her long‐held skepticism and motivated her to continue both laser and psychotherapy. Diagnostic tests included Y‐BOCS (Yale–Brown Obsessive Scale) for OCD severity and HADS (Hospital Anxiety and Depression Scale) for anxiety and depression. The patient demonstrated a significant reduction in obsessive‐compulsive symptomatology, with Y‐BOCS scores decreasing from 28 to 9, corresponding to clinically significant improvement and cessation of excoriation behavior. Psychological distress markedly decreased after successful psychodermatological therapy. Anxiety (HADS‐A): decreased from 13 to 5; Depression (HADS‐D): decreased from 10 to 4.

**FIGURE 1:**
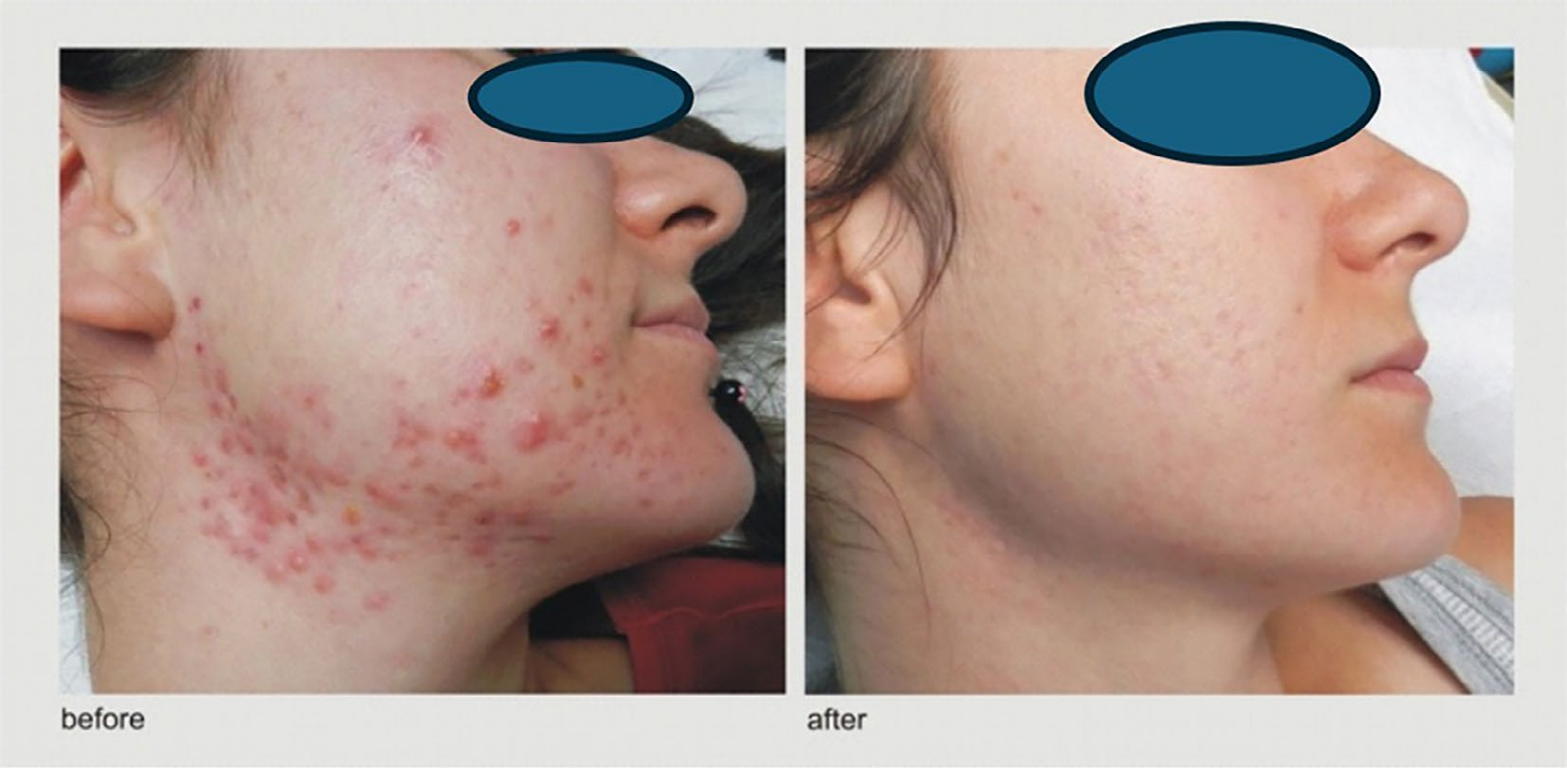
Before and “After” Therapy with the Described Psychodermatological Treatment Approach (pretreatment photo date: October 14, 2014 and post treatment photo date: June 29, 2015).

Three psychotherapy sessions used CBT for behavior modification, mindfulness for impulse control, and Habit Reversal Training for awareness, alternative behaviors, and self‐monitoring [6]. The patient was told to touch lesions only on the untreated side, helping her control excoriation urges by comparing both sides. The first three laser sessions were 2 weeks apart, then every 4–6 weeks for 6 months. One year after the last session, no excoriation or active acne remained, only residual scars. She was advised not to manipulate new lesions and reported improved self‐esteem and social confidence (Figure 1).

### 2.4 Case Report 2

A 22‐year‐old man with chronic acne reported long‐standing habits of squeezing lesions since adolescence, despite prior antibiotics, exfoliants, retinoids, and regular pimple extractions by a beautician. He noticed temporary summer improvement, likely from tanning masking erythema, with worsening in late summer due to increased sebum production. Years of unsuccessful treatments led to distrust of dermatologists and beauticians, with shame and anxiety intensifying his self‐excoriation. Examination showed no comedones, only papules, pustules, and marked post‐inflammatory erythema. Combined psychological and laser therapy began with unilateral ablative drainage of papules and pustules. At 2 weeks, he reported dramatic improvement on the treated side but continued manipulating lesions on the untreated side. Motivated to change, he requested immediate treatment for the other side, though persistent urges required two more sessions for new cysts.

**FIGURE 2:**
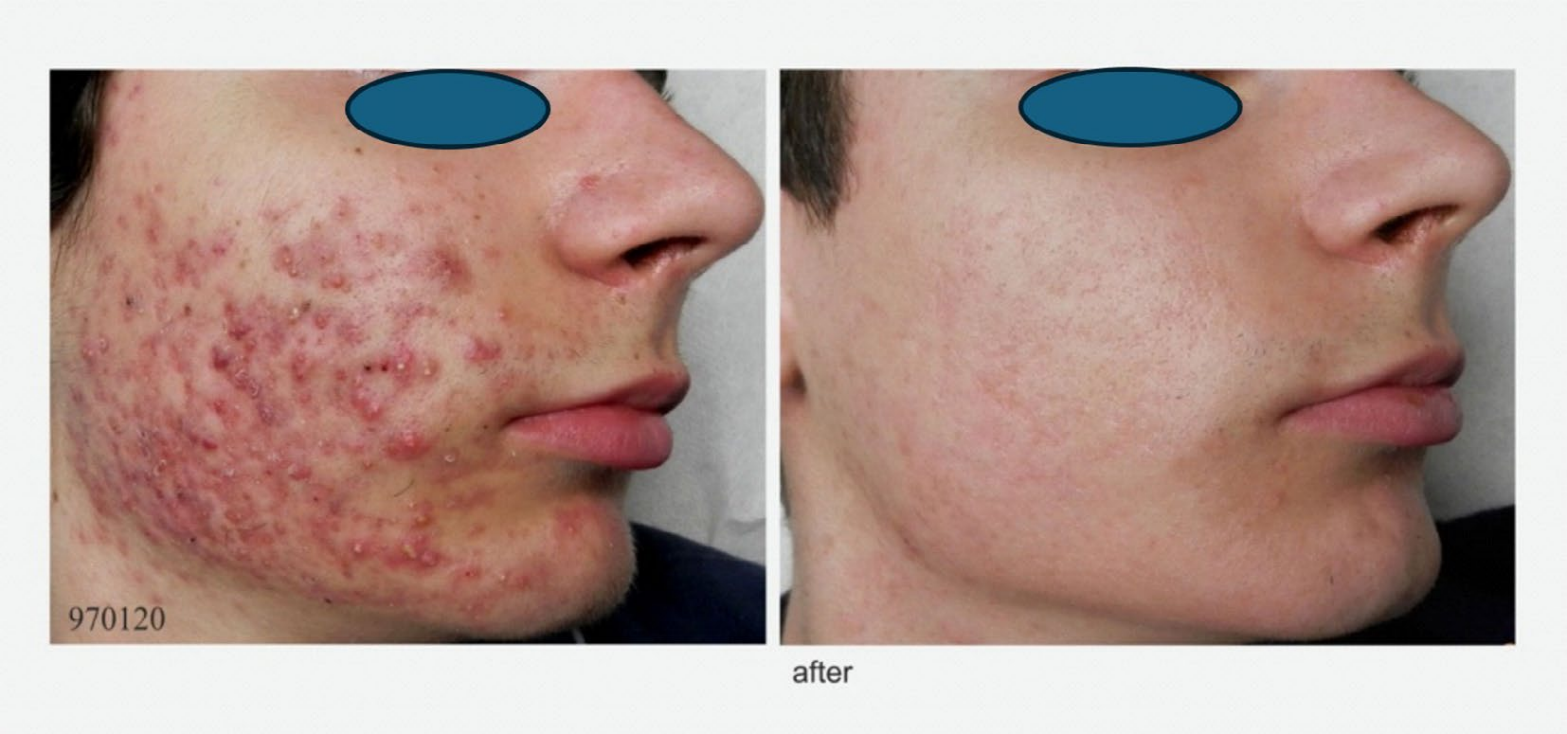
Before and “After” Therapy with the Described Psychodermatological Treatment Approach (pretreatment photo date: April 29, 2014 and Post treatment photo date: September 3, 2014).

Three psychotherapy sessions were conducted using cognitive–behavioral therapy (CBT), exposure & response prevention (ERP), emotion regulation techniques, and habit reversal therapy (HRT). After 3 weeks, marked improvement on the treated side led the patient to request treatment for the other side. He reported stopping self‐excoriation, and with no signs detected, laser therapy began contralaterally. Two months later, only fading post‐inflammatory pigmentation remained. He maintained cessation of excoriation, improved well‐being, and increased social activities, with 2 more months of maintenance psychotherapy to prevent relapse (Figure 2).

### 2.5 Case Report 3

A 40‐year‐old woman had over 10 years of mandibular and chin acne with cysts, atrophic scars, and no comedones. She claimed to have tried “everything possible” per various specialists, cleansed with antimicrobial products, used numerous cosmetics, and applied heavy makeup. She feared microbes and believed her acne was due to “blood poisoning.” Symptoms fluctuated but worsened with stress. Examination showed papules, pustules, and excoriation marks. She admitted to frequent touching and squeezing for temporary relief, concealing inflammation with makeup. Referred for psychiatric evaluation due to her belief about “blood poisoning,” causing her acne. She categorically refused, stating “I am not crazy”. She declined both consultation and psychological assessment instruments.

**FIGURE 3:**
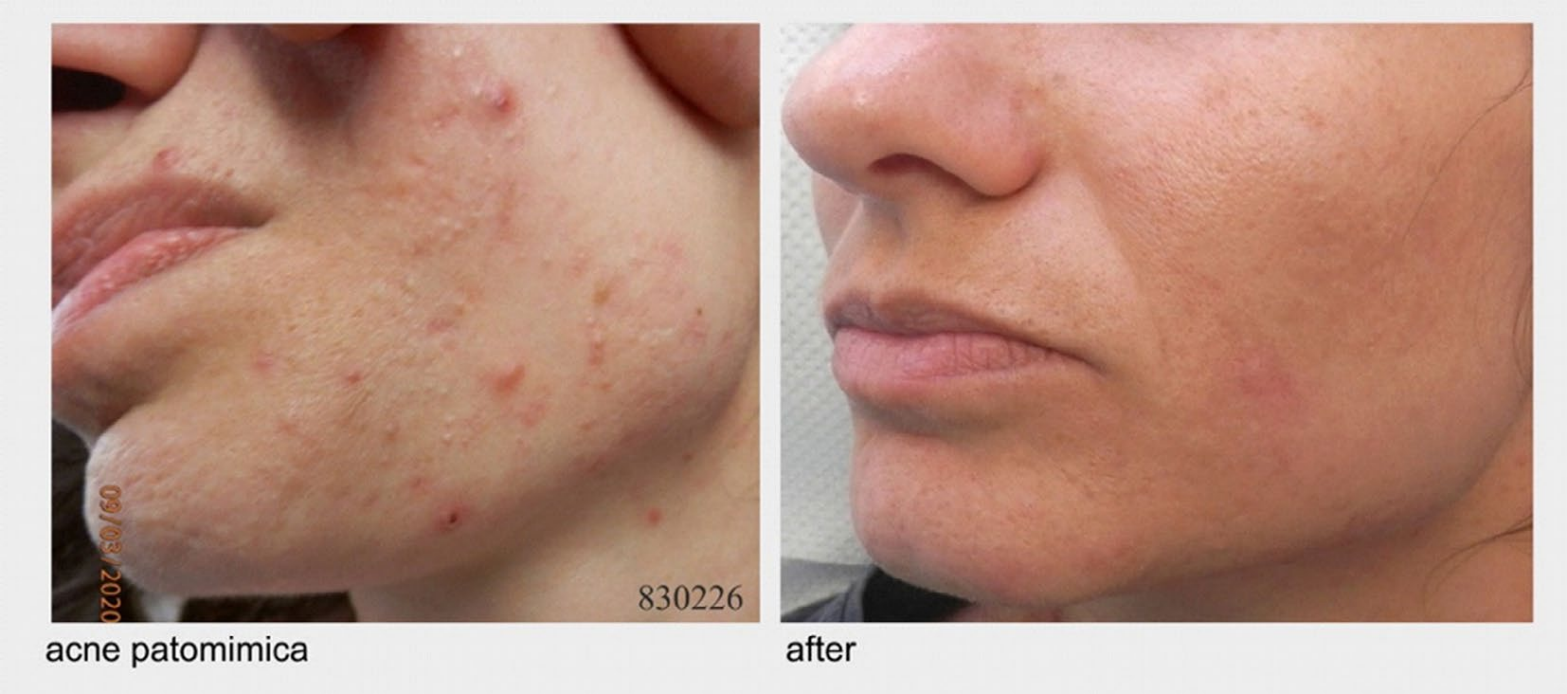
Before and “After” Therapy with the Described Psychodermatological Treatment Approach (Pre‐treatment photo date: March 9, 2020 and Post treatment photo date: May 18, 2020).

The same psychodermatological approach as in previous cases was used, combining laser cyst evacuation with an integrative psychotherapy plan. CBT targeted anxiety, OCD symptoms, and irrational beliefs, while ERP addressed triggers for picking. Over 3 months, the patient underwent six laser and three psychotherapy sessions, resulting in marked acne clearance, resolution of microbial and “blood poisoning” fears, and cessation of self‐aggressive behavior (Figure 3).

## 3 Results

In all three cases, treatment combined psychobehavioral therapy for compulsive skin‐picking with precision laser‐assisted sebaceous gland ablation using a 250‐μm super pulse beam at 3–4 W. This low energy was sufficient to coagulate the gland and facilitate evacuationof its contents with minimal thermal damage of the surrounding tissues. Initial unilateral laser sessions produced visible improvement, reducing excoriation urges and enhancing psychotherapy effectiveness, resulting in rapid, clinically significant outcomes.

## 4 Discussion

Although acne is multifactorial, the psychobehavioral component remains overlooked and neglected by both dermatologists and patients. In acne excoriée, self‐inflicted skin trauma from compulsive picking causes sebaceous gland outlet epithelialization and cyst formation. This phenomenon is most clinically significant in acne excoriée, wherein patients exhibit repetitive, even ritualistic picking behaviors (e.g., squeezing and scratching). Affected individuals may spend hours focused on skin examination and manipulation. They pick, squeeze, scratch, and manipulate real (or perceived) acne lesions, which leads to worsened inflammation, scarring, and further psychosocial distress. This behavior persists despite various negative consequences (scarring, infections, shame, etc.) and is strongly associated with body‐focused repetitive behaviors (BFRBs). Classified in DSM‐5 as Excoriation Disorder, it involves repetitive squeezing or scratching, sometimes for hours, worsening inflammation, scarring, and distress. This behavior, linked to BFRBs, often coexists with anxiety, OCD, and depression, serving as maladaptive coping [12], [13], [10]. Although patients with these acne presentations commonly have psychiatric comorbidities, they are rarely referred to integrated dermatology–psychiatry clinics [6]. The literature indicates that adult women with acne excoriée may represent a distinct clinical population that is frequently refractory to standard dermatologic therapies as compulsive picking often persists for years following acne resolution [11]. Misconceptions, such as believing squeezing cleanses skin, reinforced by beautician practices further perpetuate the habit, making targeted behavioral intervention essential for progress.

Current acne management employs various therapeutic approaches, such as antibiotics (tetracyclines and macrolides) targeting Cutibacterium acnes and inflammation; retinoids (topical adapalene; oral isotretinoin) directed at keratinization and sebum production, hormonal therapies and anti‐inflammatory agents (corticosteroids, etc.) for papulopustular lesions; photodynamic and laser therapies (PDL, KTP, and IPL) focusing on suppression of sebaceous secretion and erythema. Natural compounds from plants are also being studied as acne treatments, targeting bacteria, clogged pores, and inflammation [11].

Isotretinoin is currently considered as one of the most effective treatments for severe acne when properly managed, [14, 15, 16, 17, 18] yielding satisfactory results despite a range of potential side effects [16, 17, 18, 19]. Light and laser therapies (PDL pulsed dye laser), KTP (Potassium Titanyl Phosphate), and IPL (intense pulsed light) have gained popularity recently, with medical literature documenting their use in acne treatment [20, 21, 22, 23].

While PDL may help with post‐lesional vascularization, this usually resolves on its own. KTP and IPL might worsen acne by raising tissue temperature and triggering inflammation. Benefits from these light therapies may reflect acne's natural cycles or placebo effects rather than direct treatment impact. Claims that specific wavelengths like the yellow light spectrum (577–578 nm), PDL, or copper bromide lasers effectively target porphyrins (the metabolic byproducts of these microorganisms that release free oxygen to suppress *Cutibacterium acnes*) are debatable, especially since reducing *C. acnes* can encourage *Staphylococcus aureus* growth, potentially worsening acne. Laser treatments show promise but need larger, long‐term trials to confirm effectiveness, especially for sebaceous gland ablation, which hasn't been proven [24, 25, 26]. Unlike fractional CO_2_ lasers for scars, ablative CO_2_ laser use on sebaceous glands hasn't been reported. Psychotherapeutic treatments, such as HRT and CBT, though often seen as adjuncts, have shown benefits and can improve acne excoriée, anxiety, and quality of life [12], [27], [28], [29]. Psychiatric evaluation is essential for severe or chronic skin‐picking cases [28].

Our findings show that psychobehavioral factors are the main cause of obstructed sebaceous glands leading to excoriation, with traditional factors playing a secondary role. Mechanical trauma from picking triggers inflammation and a cycle of epithelialization that blocks glands, forming cysts. Maladaptive behaviors sustain this cycle, causing prolonged, severe acne. Clinical data drawn from these three cases demonstrated rapid improvement with combined behavioral therapy and gland ablation, highlighting excoriation as the often‐overlooked key trigger in treatment‐resistant acne excoriée.

## 5 Conclusion

These three cases show that quickly controlling chronic post‐adolescent acne is possible by stopping excoriation and physically removing blocked sebaceous glands, confirming excoriation as the key but often overlooked trigger. Psychotherapy interrupts the cycle of formation of new epithelized occluded glands by stopping excoriation behavior, while laser treatment boosts patient motivation through visible results. The findings support a shift toward combined laser and psychotherapeutic approaches for effective, integrated acne care. Effective acne excoriée management requires a psychodermatological approach that addresses stress, anxiety, OCD, and body‐focused repetitive behaviors. Thorough evaluation of psychiatric comorbidities and excoriation severity should guide treatment. Future controlled studies are needed to develop standardized protocols and confirm long‐term benefits. Personalized strategies are essential as compulsive behaviors often sustain the condition.

## Ethics Statement

This manuscript reports original research, not previously published, and is not under consideration by another journal. All authors have approved the final version. It was an IRB exempt study since benign already established interventions were used.

## Consent

All subjects provided written informed consent regarding their photos and publication.

## Conflicts of Interest

The authors declare no conflicts of interest.

## Data Availability

The data that support the findings of this study are available on request from the corresponding author. The data are not publicly available due to privacy or ethical restrictions.
